# Shenlian Decoction Ameliorates LPS-Related Inflammation in db/db Mice: Coupling Network Pharmacology With Experimental Verification

**DOI:** 10.1155/jdr/3823051

**Published:** 2025-01-06

**Authors:** Yi-fan Liu, Yuan-yuan Liu, Yao Xiao, Wei-jun Huang, Rui-xi Sun, Jie Hu, Xiao-zhe Fu, Chu-xiao Tian, Qiang Fu, Jin-xi Zhao

**Affiliations:** ^1^Section II of Endocrinology & Nephropathy Department, Dongzhimen Hospital, Beijing University of Chinese Medicine, Beijing, China; ^2^Affiliated Hospital of Shandong University of Traditional Chinese Medicine, Shandong University of Traditional Chinese Medicine, Jinan, China; ^3^Institute of Chinese Medical Literature and Culture, Shandong University of Traditional Chinese Medicine, Jinan, China; ^4^Nephropathy Department, Beijing University of Chinese Medicine Third Affiliated Hospital, Beijing University of Chinese Medicine, Beijing, China; ^5^Key Laboratory of Chinese Internal Medicine of Ministry of Education and Beijing, Dongzhimen Hospital Affiliated to Beijing University of Chinese Medicine, Beijing University of Chinese Medicine, Beijing, China; ^6^Institute of Basic Theory, China Academy of Chinese Medical Sciences, Beijing, China

**Keywords:** inflammation, LPS, network pharmacology, Shenlian decoction, Type 2 diabetes

## Abstract

**Background:** Shenlian (SL) decoction, a renowned traditional Chinese formula for diabetes mellitus, has also been employed to treat intestinal disorders. Previous studies have demonstrated the efficacy of SL decoction in regulating blood glucose and intestinal bacteria. Nevertheless, further analysis is required to elucidate the mechanistic link between SL decoction–mediated improvement of intestinal function and treatment of Type 2 diabetes mellitus (T2DM).

**Methods:** Firstly, the active ingredients of SL decoction were sourced from the Traditional Chinese Medicine System Pharmacology (TCMSP) database, with putative targets of active ingredients being predicted using the same database. Secondly, the Online Mendelian Inheritance in Man (OMIM) and GeneCards databases were employed to screen the aforementioned targets that act on T2DM, and protein–protein interaction (PPI) networks were constructed in accordance with the results. Thirdly, Gene Ontology (GO) and Kyoto Encyclopedia of Genes and Genomes (KEGG) enrichment analyses were conducted using the Database for Annotation, Visualization, and Integrated Discovery (DAVID), which resulted in a comprehensive analysis of the association between SL decoction for the treatment of T2DM and the modulation of intestinal functions. Finally, the effect of the SL decoction on predicted lipopolysaccharide (LPS)-related targets, as well as intestinal function markers, was validated through in vivo experimentation.

**Results:** A total of 36 active ingredients and 145 potential targets of SL decoction were predicted. GO enrichment analysis indicated that the principal biological processes by which the SL decoction acted against T2DM were responses to LPSs, while KEGG enrichment analysis identified the nuclear factor kappa B (NF-*κ*B) signaling pathway and toll-like receptor signaling pathway as the key pathways involved. The in vivo experiments showed that SL decoction improved glycolipid metabolism indexes, inflammatory factor levels, and LPS levels in db/db mice. The immunohistochemical results demonstrated that the SL decoction restored the expression of Occludin, Claudin-1, and ZO-1 in the intestine and inhibited the expression of toll-like receptor 4 (TLR4), myeloid differentiation primary response gene 88 (MYD88), and NF-*κ*B in both the intestine and pancreas. Furthermore, it may influence the levels of short-chain fatty acids (SCFAs) in feces.

**Conclusions:** This research investigated the multigene pharmacological mechanism of SL decoction against T2DM using network pharmacology and in vivo experiments. SL decoction treatment of T2DM may reverse inflammation by inhibiting LPS-related pathway activation and improving intestinal function.

## 1. Introduction

Diabetes mellitus is a chronic metabolic disorder characterized by dysfunction of pancreatic *β*-cells leading to elevated glucose levels, lipid metabolism disorders, and systemic inflammation. It can cause tissue and organ damage via various mechanisms [1, 2]. Type 2 diabetes mellitus (T2DM), which accounts for more than 90% of all diabetes cases, exhibits a rising global prevalence, particularly among middle-aged and elderly individuals [3].

In recent years, the role of the intestinal system in the development of T2DM has been increasingly recognized. Numerous animal studies provide compelling evidence that intestinal inflammation and defective barrier function contribute to metabolic endotoxemia [4, 5]. Metabolic endotoxemia, marked by increased plasma lipopolysaccharide (LPS) concentration from intestinal bacteria, triggers low-grade systemic inflammation, exacerbating obesity, insulin resistance, and diabetes progression. This can result in the onset of low-grade, systematic, and tissue inflammation, which in turn induces obesity and insulin resistance and further exacerbates diabetes [6, 7].

Traditional Chinese medicine (TCM) shows promise as an alternative treatment for diabetes. A systematic review and meta-analysis found that combining TCM with Western medicine significantly reduced fasting blood glucose (FBG) and 2-h postprandial plasma glucose (2hPG) levels while improving clinical efficacy compared to Western medicine alone [8]. TCM also shows the potential to preserve intestinal barrier function. According to reports, treatment with the Ge-Gen-Qin-Lian decoction adjusts the structure of the general intestinal microbiota, reduces intestinal inflammation, and reduces serum glucose and levels of proinflammatory cytokines [9]. Ganluyin improves dextran sulfate sodium (DSS)-induced ulcerative colitis by enhancing the expression of tight junction proteins, such as Occludin, Claudin-1, and ZO-1, while inhibiting the enteric-origin toll-like receptor 4 (TLR4)/nuclear factor kappa B (NF-*κ*B) pathway [10].

The Shenlian (SL) decoction, consisting of Renshen (*Panax ginseng* C.A.Mey.) and Huanglian (*Coptis chinensis* Franch), was first described for diabetes treatment in the Tang Dynasty book *Qianjin Fang* by Sun Simiao. In the Ming dynasty, these two herbs were originally prescribed and documented as SL decoction in the book *Wanbinghuichun*, where they were used to treat intestinal disorders. Recent studies have highlighted the benefits of Renshen and Huanglian in improving glucose metabolism and enhancing intestinal function [11–14]. In our previous study [15], we confirmed that the hypoglycemic effects of SL decoction are linked to the modulation of intestinal microbiota, which also influences inflammation. LPS is a crucial mediator of intestinal function and systemic inflammation. This study was aimed at investigating whether the mechanism of action of SL decoction in treating T2DM involves LPS, using network pharmacology and supporting this hypothesis through animal experimentation.

## 2. Materials and Methods

### 2.1. Collection and Selection of Active Ingredients From SL Decoction and Prediction of Their Targets

The primary active ingredients of SL decoction were sourced from the Traditional Chinese Medicine System Pharmacology (TCMSP) database (https://old.tcmsp-e.com/tcmsp.php). Screening criteria from the TCMSP database were established with thresholds set at oral bioavailability (OB) ≥ 30% and drug − like properties (DL) ≥ 0.18 [16]. The TCMSP database was employed to identify and predict the relevant targets of the ingredients in SL decoction. Target genes and proteins were subsequently standardized by querying the UniProtKB search page of the UniProt database (https://www.uniprot.org/) using humans as the specified species. Targets related to T2DM were obtained from two databases: The GeneCards database (https://www.genecards.org/) provides comprehensive information on human genes, searched using the keyword “type 2 diabetes mellitus” and further filtered by relevance score > 1. The Online Mendelian Inheritance in Man (OMIM) database (http://www.omim.org/, updated April 20, 2024) is continually updated, focusing on human Mendelian genetic disorders and searching using the term “type 2 diabetes mellitus”. The OMIM database was searched using the search term “type 2 diabetes mellitus”, and the resulting targets were pooled and duplication removed with the GeneCards database results using the VENN online tool (http://bioinformatics.psb.ugent). The overlapping targets were calculated in the meantime.

### 2.2. Protein–Protein Interaction (PPI) Data and Network Construction

PPI networks were constructed using the STRING database (accessible via https://cn.string-db.org/) to identify potential targets for treating T2DM with SL decoction [17]. The overlapping targets of SL decoction and T2DM were imported into the STRING database, and the following conditions were set for the subsequent analysis: biological species as *Homo sapiens* and the minimum interaction threshold as “medium confidence ≥ 0.9, except for text mining.” A PPI network comprises nodes representing target genes, edges between nodes representing interactions between these genes, and a “degree” value for each node indicating the number of edges connected to it. Tab-separated value (TSV) files were then imported into R software, and the number of protein interaction relationships in the network was computed using the provided script to identify hub genes. Finally, hub genes were ranked by their degree values to pinpoint core targets.

### 2.3. Gene Ontology (GO) and Kyoto Encyclopedia of Genes and Genomes (KEGG) Enrichment Analyses

GO and KEGG enrichment analyses were performed using the Database for Annotation, Visualization, and Integrated Discovery (DAVID) (https://david.ncifcrf.gov/). DAVID integrates diverse biological databases and analytical tools to elucidate the biological significance of specific genes or proteins from large-scale gene or protein sequences. The core targets identified for SL decoction in T2DM were input into DAVID for these analyses. The results were then visualized using the bioinformatics online tool (https://www.bioinformatics.com.cn/).

### 2.4. Experimental Validation In Vivo

#### 2.4.1. Drugs and Reagents

Renshen and Huanglian were procured from Beijing Kangmei Pharmaceutical Co. Ltd., with chemical compositions consistent with our previous reports [15]. Metformin (Met) hydrochloride tablets were provided by Sino-American Shanghai Squibb Pharmaceuticals Ltd.

#### 2.4.2. Animal

Animal experiments were conducted following guidelines approved by the Animal Welfare Committee of Beijing University of Chinese Medicine (Approval No. 21-19). Six-week-old male C57BL/KsJ-db/db mice and their normal littermates (db/m) were procured from Changzhou Cavens Experimental Animal Co. Ltd. (Approval Number: SCXK (su) 2016-0010). The animals were housed in a facility with temperatures ranging from 20°C to 26°C and a daily variation not exceeding 3°C. The humidity level in the facility was maintained between 40% and 70%, and a 12-h light–dark cycle was maintained. Animals were provided ad libitum access to a standard chow diet and water.

#### 2.4.3. Experiment Design

db/db mice were randomly assigned to three groups (*n* = 6): the diabetic control (DC) group received distilled water (0.2 mL/10 g), the SL group received SL decoction (combination of Huanglian and Renshen, 4.55 and 0.455 g/kg, respectively [15]), and the Met group received Met (0.228 g/kg). Normal control (NC) mice consisted of db/m mice. Interventions were administered via gavage (0.2 mL/10 g) over an 8-week period. During the final 3 days of the trial, fresh fecal samples were collected from each animal using the clean catch method, immediately transferred into sterile centrifuge tubes, and stored at −80°C. Peripheral blood was obtained from the ophthalmic vein under anesthesia induced by intraperitoneal injection of a 0.3% sodium pentobarbital solution (0.2 mL/10 g). Mice were euthanized by cervical dislocation. Blood was allowed to clot at room temperature for 1 h, followed by serum collection after centrifugation (4000 g, 10 min, 4°C). The ileum and pancreas were dissected and immersed in a 4% paraformaldehyde solution adjusted to pH 7.4 for fixation.

#### 2.4.4. Serum Parameter Assay

Glycated serum protein (GSP) reflects the average blood glucose levels over the last 2 weeks of the experiment and is more informative than a single blood glucose measurement. GSP, triglycerides (TGs), and total cholesterol (TC) levels were measured using corresponding assay kits (Nanjing Jiancheng Bioengineering Research Institute Co. Ltd., A037-1-0, A037-1-1) following the manufacturer's instructions. LPS content was assessed using the chromogenic Limulus amebocyte lysate assay kit (GenScript, L00350). Serum levels of TGF-*β*1, IL-1*β*, IL-6, and TNF-*α* were measured using commercial ELISA kits (Elabscience, E-EL-0162, E-MSEL-M0003, E-MSEL-M0001, E-MSEL-M0002).

#### 2.4.5. Ileum Histological Morphological Observation

Ileum samples were prepared as previously described [18]. Subsequently, the slides were routinely stained with hematoxylin–eosin and then observed under an optical microscope.

#### 2.4.6. Immunohistochemistry

Immunohistochemistry was performed as previously described [19]. Antibodies used included TLR4, NF-*κ*B, myeloid differentiation primary response gene 88 (MYD88), ZO-1, Claudin-1, and Occludin (Proteintech, 19811-1-AP, 10745-1-AP, 23230-1-AP, 21773-1-AP, 13050-1-AP, 27260-1-AP). The mean density of positive proteins was quantified using ImageJ 1.51j8 software.

#### 2.4.7. Gas Chromatography–Mass Spectrometry (GC-MS) Analysis of Short-Chain Fatty Acids (SCFAs)

Fecal levels of SCFAs, including acetic acid, propionic acid, butyric acid, isobutyric acid, valeric acid, isovaleric acid, hexanoic acid, and isohexoic acid, were determined as described previously [20]. Briefly, 300 mg of fecal samples were homogenized with 1 mL of ddH_2_O and centrifuged at 14,000 g for 10 min at 4°C. The supernatant was homogenized with 25% metaphosphoric acid in a volume ratio of 1:1, incubated at room temperature for 4 h, and then centrifuged at 12,000*g* for 15 min at 4°C. The supernatant was filtered through a 45 *μ*m microporous membrane, and the final concentration of SCFAs was determined using a GC-MS system (Agilent 7890/5975C, Santa Clara, CA, United States).

#### 2.4.8. Analysis of Data

Data were presented as mean ± SEM (standard error of mean) and analyzed using SPSS 22.0. Normality was assessed with the Shapiro–Wilk *W* test, and homogeneity of variances was confirmed. One-way analysis of variance (ANOVA) analysis was performed to evaluate statistical differences between groups. Based on the results of the homogeneity of variance test, post hoc tests such as the least significant difference (LSD) or Dunnett's *T*3, assuming unequal variances, were utilized for comparing means among groups. Statistical significance was set at *p* < 0.05.

## 3. Results

### 3.1. Prediction of Active Ingredients and Therapeutic Targets for T2DM in SL Decoction

**Figure 1:**
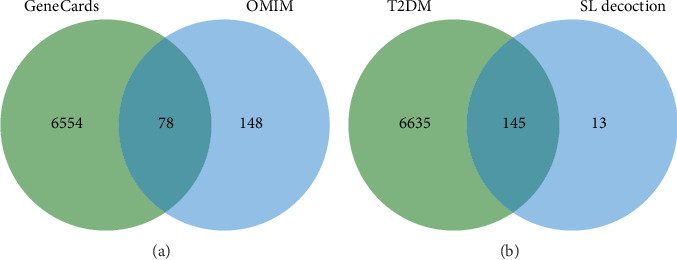
Prediction of compound-related targets and T2DM-related targets. (a) Venn diagram depicting targets identified in GeneCards and OMIM. (b) Venn diagram illustrating targets identified in T2DM and SL decoction.

A search of the TCMSP database identified 36 ingredients for SL decoction that met the criteria of OB ≥ 30% and DL ≥ 0.18. Specifically, 22 ingredients were from Renshen and 14 from Huanglian (Table 1). In previous studies, we have validated the concentrations of several key components of SL decoction [15]. Moreover, a total of 145 potential therapeutic targets for treating T2DM were predicted. GeneCards identified 6632 genes relevant to T2DM (with a relevance score greater than 1), while the OMIM database contributed 226 genes. After removing duplicates, a comprehensive set of 6780 genes associated with T2DM was compiled (depicted in Figure 1(a)). Using the Venn plot tool, it was confirmed that 145 potential targets of SL decoction were linked to T2DM (Figure 1(b)).

### 3.2. PPI Network for SL Decoction Against T2DM

**Figure 2:**
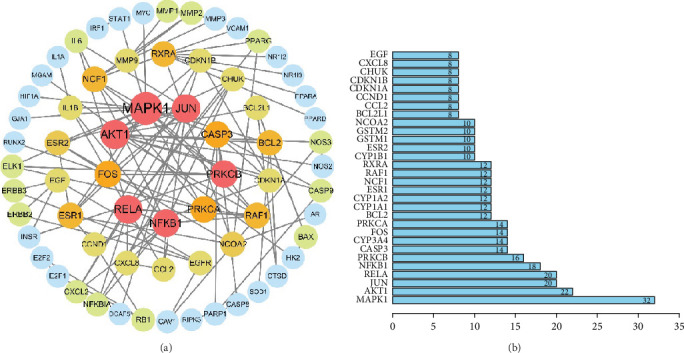
PPI network of SL decoction targets against T2DM. (a) PPI network encompassing all target genes of SL decoction against T2DM. (b) PPI network focusing on the Top 30 target genes of SL decoction against T2DM.

A new PPI network was constructed from the STRING database using genes related to SL decoction and T2DM. This network was visualized using Cytoscape (Version 3.7.0) (Figure 2(a)). The network consisted of 65 nodes and 244 edges, where node size reflected the degree of centrality determined through topological analysis. Degree values for the 30 most central genes were computed using Cytoscape (Version 3.7.0) and are depicted in Figure 2(b). The Top 10 hub genes identified through topology analysis include MAPK1, AKT1, RELA, JUN, NFKB1, PRKCB, FOS, PRKCA, CASP3, and RXRA. These genes stand out due to their numerous interactions with other target genes.

### 3.3. GO and KEGG Pathway Enrichment Analyses

**Figure 3:**
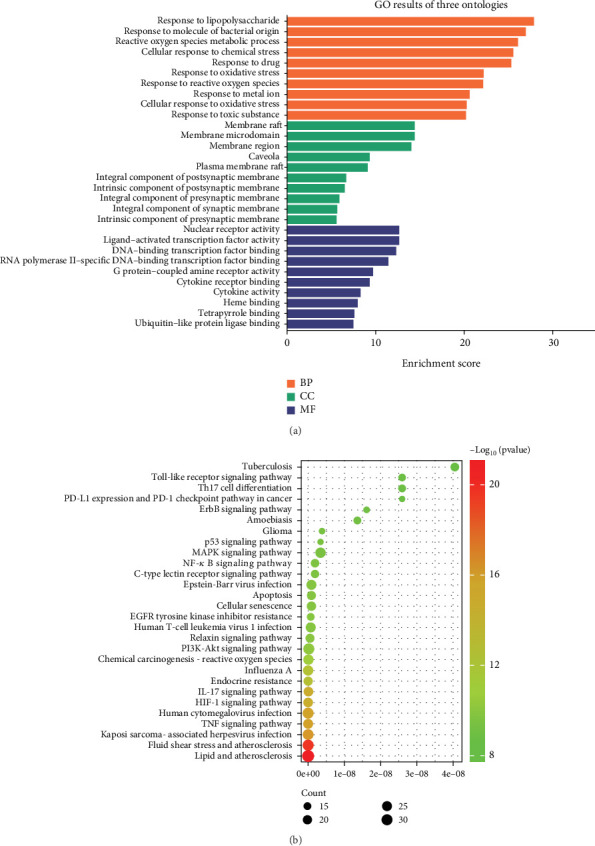
Findings from biological processes and pathway enrichment analysis. (a) Results of GO enrichment analysis for potential targets of SL decoction in T2DM. (b) Outcomes of KEGG pathway enrichment analysis for potential anti-inflammatory targets of SL decoction in T2DM. The *q*-value in each case corresponds to the −log10 *p* value.

A GO analysis was performed to explore the biological functions of the 145 targets of SL decoction in T2DM. The GO enrichment analysis revealed that these targets predominantly localize in membrane rafts, microdomains, caveolae, and other cellular regions. They are also involved in responses to LPSs, oxidative stress, chemical stress, and drugs. Additionally, they exhibit molecular functions such as nuclear receptor activity, ligand-activated transcription factor activity, DNA-binding transcription factor binding, cytokine receptor binding, and cytokine activity, highlighting their roles in combating T2DM (Figure 3(a)).

To further elucidate the potential anti-inflammatory mechanism of SL decoction in T2DM, a KEGG pathway enrichment analysis was conducted on the 145 targets. The Top 30 pathways associated with inflammation were identified based on their *p* values (Figure 3(b)). Key pathways implicated in mediating inflammation include the TNF signaling pathway, IL-17 signaling pathway, NF-*κ*B signaling pathway, Th17 cell differentiation, and toll-like receptor signaling pathway [21, 22].

In conclusion, the therapeutic effect of SL decoction on T2DM appears closely linked to nuclear receptor activity, response to LPSs, and response to molecules of bacterial origin, particularly within membrane rafts and microdomains. Among the key pathways identified, the NF-*κ*B signaling pathway and the toll-like receptor signaling pathway can be activated by LPSs, with interactions observed between these pathways. Furthermore, pivotal targets identified from the PPI network, such as NFKB1, IL-6, and IL1B, are also involved in these pathways. Therefore, in this study, the key proteins within these pathways were selected and experimentally validated to further investigate the anti-inflammatory mechanism of SL decoction in treating T2DM. It should be noted that although the PPI network also highlights the importance of genes such as AKT and MAPK1 in the anti-inflammatory mechanism of SL decoction, the results of GO and KEGG pathway enrichment analyses suggested that SL decoction may have greater potential in ameliorating LPS-induced inflammation. The relevant validation experiments linked the modulation of intestinal barrier function with the improvement of pancreatic inflammation.

### 3.4. SL Decoction Improved Serum Glucose and Lipid Levels in db/db Mice

**Figure 4:**
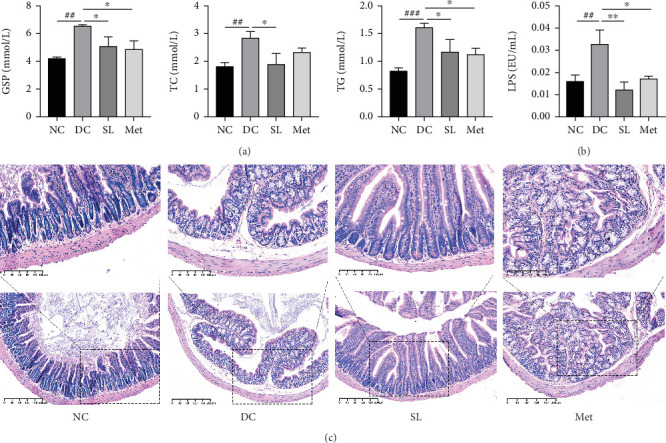
SL decoction regulated glycolipid metabolism, improved intestinal histopathology, and reduced the passage of LPS through the intestinal barrier. (a) SL decoction decreased serum levels of GSP, TG, and TC in db/db mice. (b) SL decoction improved LPS-related metabolic endotoxemia, reflecting the restoration of intestinal barrier function. (c) Representative images of HE staining of ileum samples from each group (scale bar, 100 *μ*m). Significant differences were denoted as ^##^*p* < 0.01 and ^###^*p* < 0.001 compared to the NC group; ⁣^∗^*p* < 0.05 and ⁣^∗∗^*p* < 0.01 compared to the DC group, with *n* = 6.

The impact of SL decoction on GSP, TG, and TC levels was assessed. As depicted in Figure 4(a), a significant increase in GSP levels was observed in the DC group compared to the NC group. Both the SL and Met groups exhibited a notable decrease in GSP levels, indicating lower average blood glucose levels during the final 2 weeks of the intervention. Lipid levels were evaluated by measuring TC and TG. TG and TC levels showed significant increases in the DC group, which were mitigated by SL decoction treatment. Met, on the other hand, significantly reduced only the TG level.

### 3.5. SL Decoction Ameliorated Pathological Ileal Injury and Restored Intestinal Barrier Function

**Figure 5:**
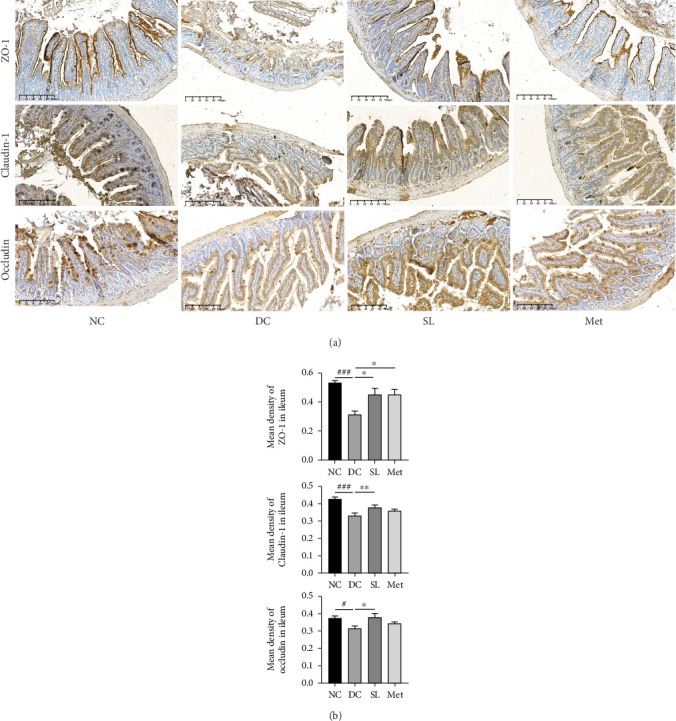
SL decoction improved intestinal permeability and enhanced expression of intestinal tight junction proteins. (a) Representative images illustrating immunohistochemical staining for ZO-1, Claudin-1, and Occludin in ileum samples from each experimental group are provided (scale bar, 100 *μ*m). (b). Statistical analysis of immunohistochemistry results is depicted in column charts. Significant differences were denoted as ^#^*p* < 0.05 and ^###^*p* < 0.001 compared to the NC group; ⁣^∗^*p* < 0.05 and ⁣^∗∗^*p* < 0.01 compared to the DC group, with *n* = 4.

HE staining of ileum tissue revealed that the DC group exhibited defects in intestinal tight junctions, including villi edema, increased villi width, and extended distance between the base of villous crypts and the muscularis propria. In contrast, treatment with SL decoction prevented tissue damage in the ileum (Figure 4(c)). The levels of tight junction molecules, such as Occludin, Claudin-1, and ZO-1, are crucial for intestinal permeability. Protein expression of Occludin, Claudin-1, and ZO-1 was significantly reduced in the DC group but significantly restored with SL decoction treatment (Figure 5). Met also improved ZO-1 expression. Elevated intestinal permeability in the DC group led to significantly higher serum LPS levels compared to the NC group. Both SL decoction and Met treatments effectively mitigated LPS elevation, as depicted in Figure 4(b). These results align with existing literature on Met treatment efficacy [23].

### 3.6. SL Decoction Remarkably Inhibited Metabolic Inflammation by Suppressing the LPS/TLR4/MYD88/NF-*κ*B Pathway

**Figure 6:**
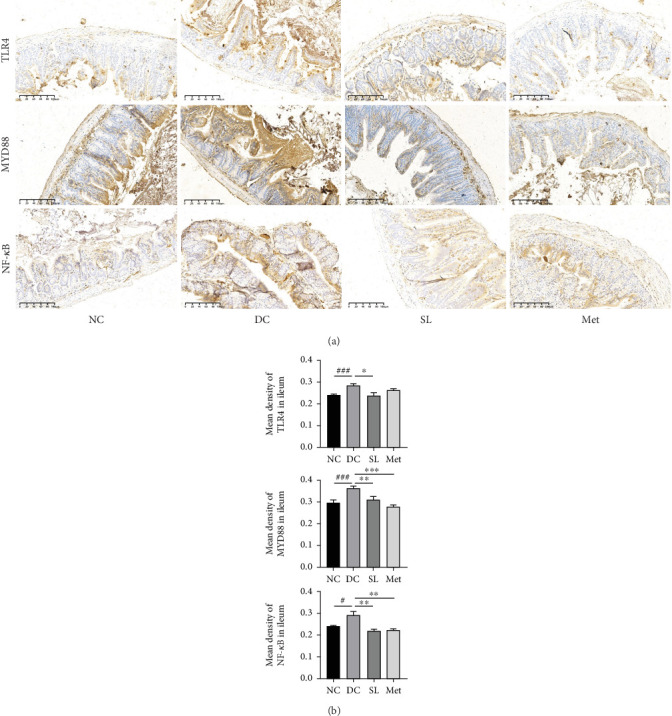
SL decoction inhibited ileum inflammation through modulation of the TLR4/MYD88/NF-*κ*B pathway. (a) Representative images illustrating immunohistochemical staining for TLR4, MYD88, and NF-*κ*B in ileum samples from each experimental group are provided (scale bar, 100 *μ*m). (b) Statistical analysis of immunohistochemistry results is depicted in column charts. Significant differences were denoted as ^#^*p* < 0.05 and ^###^*p* < 0.001 compared to the NC group; ⁣^∗^*p* < 0.05 and ⁣^∗∗^*p* < 0.01 compared to the DC group, with *n* = 4.

**Figure 7:**
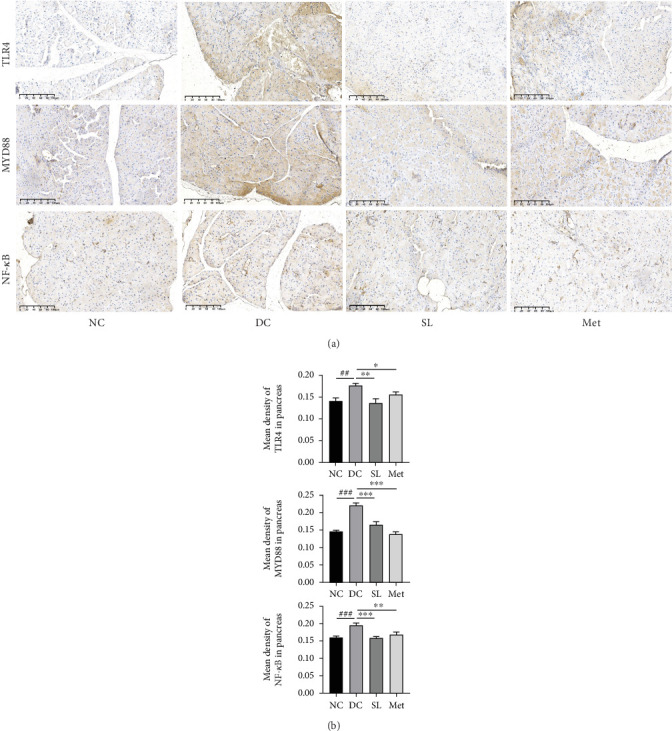
SL decoction inhibited pancreatic inflammation through modulation of the TLR4/MYD88/NF-*κ*B pathway. (a) Representative images illustrating immunohistochemical staining for TLR4, MYD88, and NF-*κ*B in pancreas samples from each experimental group are provided (scale bar, 100 *μ*m). (b) Statistical analysis of immunohistochemistry results is depicted in column charts. Significant differences were denoted as ^##^*p* < 0.01 and ^###^*p* < 0.001 compared to the NC group; ⁣^∗^*p* < 0.05 and ⁣^∗∗∗^*p* < 0.001 compared to the DC group, with *n* = 4.

**Figure 8:**
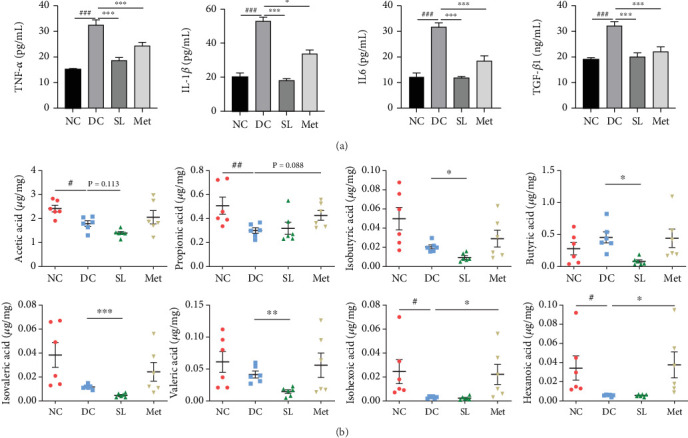
Effects of SL decoction on inflammatory factors and SCFAs. (a) SL decoction decreased serum levels of TNF-*α*, IL-1*β*, IL-6, and TGF-*β*1. (b) SL decoction reduced the levels of several SCFAs, including isobutyric acid, butyric acid, isovaleric acid, and valeric acid. Significant differences were denoted as ^#^*p* < 0.05 and ^###^*p* < 0.001 compared to the NC group; ⁣^∗^*p* < 0.05 and ⁣^∗∗^*p* < 0.01 compared to the DC group, with *n* = 6.

The presence of LPS in serum activated the TLR4/MYD88/NF-*κ*B pathway, leading to chronic inflammation across multiple systems, including not only the ileum but also the pancreas. As illustrated in Figures 6 and 7, increased activation of TLR4, MYD88, and NF-*κ*B was observed in the ileum of the DC group, paralleled by similar increases in the pancreas. Treatment with SL decoction or Met effectively suppressed the activation of the TLR4/MYD88/NF-*κ*B pathway in both organs. Additionally, serum levels of inflammatory factors TNF-*α*, IL-1*β*, IL-6, and TGF-*β*1 were significantly elevated in the DC group (Figure 8(a)). However, these elevations were attenuated in the SL and Met groups [24].

### 3.7. SL Decoction Reduced Fecal Levels of SCFAs

The study investigated fecal levels of SCFAs. As shown in Figure 8(b), most types of SCFAs were reduced to some extent in the DC group compared to the NC group. Specifically, acetic acid, propionic acid, hexanoic acid, and isohexoic acid showed statistically significant reductions. The SL group exhibited lower levels of isobutyric acid, butyric acid, isovaleric acid, and valeric acid, indicating a trend towards reducing SCFA levels. In contrast, Met significantly increased the levels of propionic acid, hexanoic acid, and isohexoic acid, suggesting an increase in SCFAs.

## 4. Discussion

LPS-mediated inflammation is a critical pathological mechanism in the development of T2DM, exacerbated by intestinal barrier damage, a factor often overlooked in current therapeutic strategies. Hence, therapeutic strategies should address inflammation by safeguarding intestinal barrier integrity. According to Chinese medicine theory, T2DM is termed “emaciation-thirst disease,” attributed to qi deficiency, yin deficiency, and heat toxicity. Qi deficiency reflects a state of weakness, identified as fatigue and labored breathing; yin deficiency reflects a state of dehydration or difficulty in achieving tranquility, identified as dry skin and mouth, and heat vexation fever accompanied by restlessness; heat toxicity is associated with infectious or inflammatory states [25]. A single herbal formula can treat multiple diseases, a concept known as homotherapy for heteropathy. The SL decoction was historically used in ancient China to treat both diabetes mellitus and gastroenteritis. Renshen benefits qi and nourishes yin, while Huanglian clears heat and detoxifies toxins. These two herbs work in tandem with each other. Studies have shown that Huanglian exhibits anti-infective and anti-inflammatory effects, while Renshen regulates immune function [26, 27]. These two herbs synergistically treat inflammation. Our research has shown that SL decoction regulates intestinal bacteria, thereby influencing LPS production. However, the specific anti-inflammatory mechanism requires further exploration and validation. SL decoction's multicomponent and multitarget properties prompted this study, which uses a network pharmacology approach to elucidate its potential mechanisms against inflammation and to focus on LPS-related molecular pathways.

We screened 36 active ingredients and 145 potential targets for T2DM by applying the TCMSP database according to established methods [16]. Berberine, one of the primary active ingredients in SL decoction, exhibits multiple biological activities, including regulation of glucose and lipid homeostasis, anti-inflammatory, antioxidant, anticancer properties, and protection of the intestinal barrier [28]. Quercetin, a flavonoid compound, exhibits antihyperglycemic, cholesterol-lowering, and antioxidant properties. It also promotes *β*-cell regeneration and insulin release in pancreatic cells [29]. Ginsenoside Rg5 (Rg5) is a minor ginsenoside produced during the steaming of Renshen. It demonstrates superior pharmaceutical activity compared to major ginsenosides. Generally, Rg5 has exhibited significant potential as a broad-spectrum anticancer and anti-inflammatory agent. Additionally, Rg5 has been shown to possess antidiabetic, antiobesity, cardioprotective, and neuroprotective properties [30]. In summary, these active ingredients from the SL decoction exhibit significant potential for anti-inflammatory effects.

The results of the GO enrichment analysis revealed that SL decoction primarily affects biological processes related to responses to LPSs and bacterial molecules. This finding aligns with SL decoction's role in modulating intestinal bacteria. LPS, a hallmark metabolite indicating pathogenicity in the intestinal tract, is predominantly produced by Gram-negative bacteria [31]. When intestinal barrier function is compromised, excessive LPS enters the bloodstream and binds to CD14, forming a complex that binds to the TLR4 receptor. This process activates the nuclear transcription factor-*κ*B (NF-*κ*B) via MYD88, promoting the synthesis and secretion of inflammatory factors such as IL-6, IL-1*β*, and TNF-*α* [32]. The results of the KEGG enrichment analysis suggested that the NF-*κ*B signaling pathway and the toll-like receptor signaling pathway are key pathways targeted by SL decoction. These pathways involve core targets identified in the PPI network, such as NFKB1, IL-6, and IL1B.

Intestinal barrier dysfunction is considered a significant factor contributing to inflammation in various diseases, including diabetes mellitus. This dysfunction results in increased intestinal permeability, allowing antigens, toxins, and pathogens from the gut lumen to penetrate the mucosal tissue, triggering inflammation. Intestinal permeability hinges on the integrity of tight junctions within the intestinal barrier. These structures, akin to “zipper-like” mechanisms, effectively seal the intercellular space, thereby preventing the infiltration of harmful substances [33]. Tight junctions primarily rely on transmembrane proteins such as ZO-1, Occludin, and claudins [34, 35]. This study was conducted using db/db mice, a widely accepted animal model for T2DM characterized by increased intestinal permeability [36]. Our findings demonstrate that SL decoction prevented ileal tissue damage, preserved the expression of Occludin, Claudin-1, and ZO-1, and decreased serum LPS levels, which showed a protective effect on intestinal barrier function. Studies have reported that ginsenosides, active ingredients in SL decoction, can ameliorate LPS-induced disruption of intestinal epithelial tight junctions [37, 38]. It is important to note that the reduction in LPS levels may also be attributed to the modulation of intestinal microbiota, in addition to the restoration of intestinal barrier integrity.

**Figure 9:**
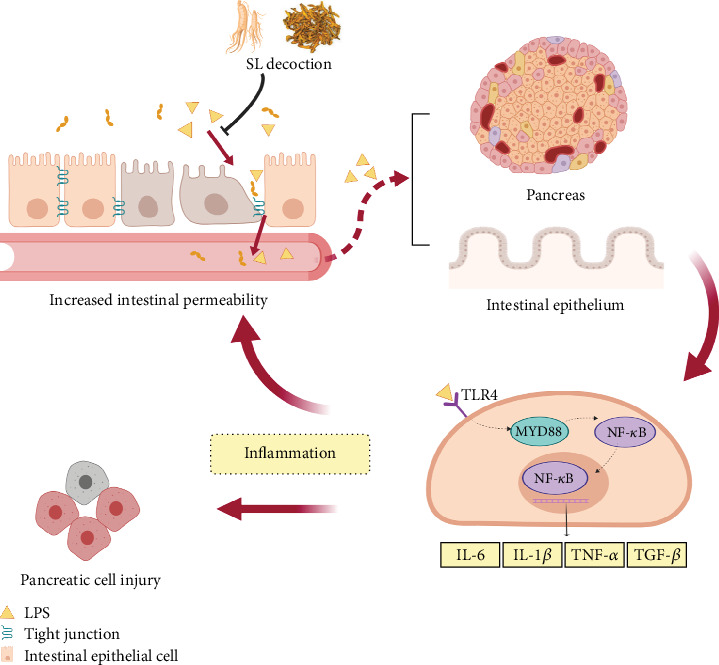
SL decoction enhances intestinal barrier function and reduces inflammation. In db/db mice, the expression of intestinal epithelial tight junction proteins, including ZO-1, Occludin, and Claudin-1, is reduced, leading to increased intestinal permeability. Consequently, toxins such as LPS can enter the circulation, activating the TLR4/MYD88/NF-*κ*B pathway in the pancreas and triggering the production of inflammatory factors. LPS also induces intestinal inflammation, exacerbating barrier dysfunction. SL decoction protects the intestinal barrier and suppresses these processes.

Low-grade inflammation is a hallmark of metabolic disorders, such as diabetes and obesity. These conditions are characterized by changes in the composition of the intestinal microbiota and their metabolites. These metabolites traverse the compromised intestinal barrier and impact various metabolic organs, including the pancreas and adipose tissue, thereby triggering metabolic inflammation [39]. Consequently, LPS exacerbates not only intestinal inflammation but also induces inflammation in the pancreas and adipocytes, contributing to impaired glucose-lipid metabolism and insulin resistance [10, 40]. Studies have reported that berberine can inhibit the TLR4/MyD88/NF-*κ*B signaling pathway, thereby ameliorating colonic inflammatory responses and intestinal epithelial barrier dysfunction in colitis mice [41]. Fermented ginseng was found to attenuate LPS-induced inflammatory responses by regulating the TLR4/MAPK signaling pathway [42]. In our previous study [15], we demonstrated that SL decoction can reverse the increased diversity of intestinal microbiota and reduce Prevotellaceae, which are associated with LPS biosynthesis. Our results consistently showed that SL decoction reduced serum LPS levels while inhibiting the activation of the TLR4/MYD88/NF-*κ*B pathway in the intestine and pancreas, thus reducing the release of inflammatory cytokines. These findings suggest that SL decoction's enhancement of the intestinal barrier promotes the suppression of chronic inflammation across various systems, potentially contributing to its role in improving glycolipid metabolism, as depicted in Figure 9.

Furthermore, the study showed that SL decoction reduced the concentration of intestinal SCFAs, which are synthesized by the intestinal microbiota from the breakdown of carbohydrates and proteins, playing crucial roles in maintaining intestinal barrier function and immune tolerance [43]. However, the implications of elevated or reduced SCFA levels in obesity and diabetes remain controversial. While many animal studies have suggested that SCFAs play a role in regulating host metabolism [44], several studies have indicated that an excess of SCFAs may adversely affect host health [43]. Overweight individuals were observed to have higher production of intestinal SCFAs compared to lean individuals [45]. Additionally, positive associations were found between higher levels of fecal acetate, propionate, and total SCFA and insulin resistance, body mass index, and fasting insulin levels in obese women [46]. Huanglian and its active components exhibit significant antibacterial effects. It has been reported in the literature that Huanglian reduces the diversity of the intestinal microbiota, while Renshen may affect SCFAs by improving the structure of the microbiota [47, 48]. Our previous research demonstrated that the diversity and richness of the intestinal microbiota in db/db mice significantly increased and could be notably inhibited by SL decoction, which may be a primary factor affecting SCFA levels [15]. Overall, the decrease in SCFAs observed in this study did not demonstrate any negative effects; therefore, in the context of diabetes, SL decoction may have beneficial effects on the intestinal microbiota and its metabolic products.

## 5. Conclusions

Specifically, enhancing intestinal barrier function and mitigating LPS-related inflammation appear to be pivotal mechanisms of SL decoction in managing T2DM. Consequently, TLR4, MYD88, and NF-*κ*B pathways activated by LPS may represent key targets of its action. These mechanisms likely contribute to SL decoction's efficacy in improving chronic inflammation across multiple systems, including the pancreas, and ameliorating disorders of glucose and lipid metabolism. Our findings also suggest a scientific basis for the historical use of SL decoction in treating diabetes and intestinal disorders. Further clinical trials in this field may complement existing treatment strategies.

## Figures and Tables

**Table 1:** Information on active ingredients of SL decoction screened by TCMSP.

**Mol ID**	**Molecule name**	**OB (%)**	**DL**	**Source**
MOL002668	Worenine	45.83	0.87	Huanglian
MOL001458	Coptisine	30.67	0.86	Huanglian
MOL002904	Berlambine	36.68	0.82	Huanglian
MOL002907	Corchoroside A_qt	104.95	0.78	Huanglian
MOL002897	Epiberberine	43.09	0.78	Huanglian
MOL001454	Berberine	36.86	0.78	Huanglian
MOL002903	(R)-Canadine	55.37	0.77	Huanglian
MOL013352	Obacunone	43.29	0.77	Huanglian
MOL002894	Berberrubine	35.74	0.73	Huanglian
MOL000785	Palmatine	64.6	0.65	Huanglian
MOL000762	Palmidin A	35.36	0.65	Huanglian
MOL000098	Quercetin	46.43	0.28	Huanglian
MOL008647	Moupinamide	86.71	0.26	Huanglian
MOL000622	Magnograndiolide	63.71	0.19	Huanglian
MOL005357	Gomisin B	31.99	0.83	Renshen
MOL000787	Fumarine	59.26	0.83	Renshen
MOL005317	Deoxyharringtonine	39.27	0.81	Renshen
MOL005376	Panaxadiol	33.09	0.79	Renshen
MOL005401	Ginsenoside Rg5_qt	39.56	0.79	Renshen
MOL005348	Ginsenoside-Rh4_qt	31.11	0.78	Renshen
MOL000449	Stigmasterol	43.83	0.76	Renshen
MOL005399	Alexandrin_qt	36.91	0.75	Renshen
MOL000358	Beta-sitosterol	36.91	0.75	Renshen
MOL005360	Malkangunin	57.71	0.63	Renshen
MOL004492	Chrysanthemaxanthin	38.72	0.58	Renshen
MOL005344	Ginsenoside rh2	36.32	0.56	Renshen
MOL005384	Suchilactone	57.52	0.56	Renshen
MOL003648	Inermin	65.83	0.54	Renshen
MOL005314	Celabenzine	101.88	0.49	Renshen
MOL002879	Diop	43.59	0.39	Renshen
MOL005321	Frutinone A	65.9	0.34	Renshen
MOL005356	Girinimbin	61.22	0.31	Renshen
MOL000422	Kaempferol	41.88	0.24	Renshen
MOL005308	Aposiopolamine	66.65	0.22	Renshen
MOL005320	Arachidonate	45.57	0.2	Renshen
MOL005318	Dianthramine	40.45	0.2	Renshen

## Data Availability

The data that support the findings of this study are available from the corresponding authors upon reasonable request.
